# Cross-cultural validation and psychometric testing of the Norwegian version of the TeamSTEPPS® teamwork perceptions questionnaire

**DOI:** 10.1186/s12913-017-2733-y

**Published:** 2017-12-02

**Authors:** Randi Ballangrud, Sissel Eikeland Husebø, Marie Louise Hall-Lord

**Affiliations:** 1 Department of Health Science Gjøvik, Faculty of Medicine and Health Sciences, Norwegian University of Science and Technology, Teknologivn. 22, 2815 Gjøvik, Norway; 2 Faculty of Health Sciences, University of Stavanger, Kjell Arholms hus, Kjell Arholms gate 43, 4021 Stavanger, Norway; 3 Department of Surgery, Stavanger University Hospital, Gerd Ragna Bloch Thorsens street 8, 4011 Stavanger, Norway; 4 Department of Health Sciences, Faculty of Health, Science and Technology, Karlstad University, Karlstad, Sweden

**Keywords:** Confirmatory factor analysis, Healthcare personnel, Teamwork, Patient safety

## Abstract

**Background:**

Teamwork is an integrated part of today’s specialized and complex healthcare and essential to patient safety, and is considered as a core competency to improve twenty-first century healthcare. Teamwork measurements and evaluations show promising results to promote good team performance, and are recommended for identifying areas for improvement. The validated TeamSTEPPS® Teamwork Perception Questionnaire (T-TPQ) was found suitable for cross-cultural validation and testing in a Norwegian context. T-TPQ is a self-report survey that examines five dimensions of perception of teamwork within healthcare settings. The aim of the study was to translate and cross-validate the T-TPQ into Norwegian, and test the questionnaire for psychometric properties among healthcare personnel.

**Methods:**

The T-TPQ was translated and adapted to a Norwegian context according to a model of a back-translation process. A total of 247 healthcare personnel representing different professionals and hospital settings responded to the questionnaire. A confirmatory factor analysis was carried out to test the factor structure. Cronbach’s alpha was used to establish internal consistency, and an Intraclass Correlation Coefficient was used to assess the test - retest reliability.

**Result:**

A confirmatory factor analysis showed an acceptable fitting model (χ^2^ (df) 969.46 (546), *p* < 0.001, Root Mean Square Error of Approximation (RMSEA) = 0.056, Tucker-Lewis Index (TLI) = 0.88, Comparative fit index (CFI) = 0.89, which indicates that each set of the items that was supposed to accompany each teamwork dimension clearly represents that specific construct. The Cronbach’s alpha demonstrated acceptable values on the five subscales (0.786–0.844), and test-retest showed a reliability parameter, with Intraclass Correlation Coefficient scores from 0.672 to 0.852.

**Conclusion:**

The Norwegian version of T-TPQ was considered to be acceptable regarding the validity and reliability for measuring Norwegian individual healthcare personnel’s perception of group level teamwork within their unit. However, it needs to be further tested, preferably in a larger sample and in different clinical settings.

**Electronic supplementary material:**

The online version of this article (10.1186/s12913-017-2733-y) contains supplementary material, which is available to authorized users.

## Background

Teamwork is integrated into today’s specialized and complex healthcare [1], and is a critical component for patient safety [2]. Furthermore, teamwork is ranked as a core competency to help improve twenty-first century healthcare services [3]. The WHO estimates that 3% to 16% of all patients are affected by adverse events while receiving hospital care [4], and that a large portion of these events are considered to be preventable [5, 6]. Research demonstrates that poor teamwork is an independent cause of many of the system failures that lead to patient harm [7–9]. Team training has been widely recognized in the patient safety literature as a method to optimize teamwork, thereby improving patient outcomes in healthcare [10–12]. Teamwork is described in terms of behaviour, cognitions and attitudes that make interdependent performance possible [13], and is defined as: “The interaction or relationship of two or more health professionals who work interdependently to provide care for patients” ([14], p. 3). In Norway, previous studies in teamwork training have focused on acute and trauma care settings [15, 16] and the effects on participants’ self-reported knowledge and confidence [17], different simulation modalities [18] and the performance of emergency teams [19]. A recent review of patient safety literature found a few Nordic, though no Norwegian studies measuring the perception of teamwork in the hospital settings [20].

In response to the importance of teamwork in improving patient safety in healthcare, the US Agency for Healthcare Research and Quality (AHRQ), in collaboration with the Department of Defense, developed the team training programme, Team Strategies and Tools to Enhance Performance and Patient Safety (TeamSTEPPS®) [21]. From 2006, TeamSTEPPS® has been the national standard for team training in US healthcare [21]. TeamSTEPPS® is an evidence-based team training programme and framework based on more than 30 years of teamwork, team training and cultural change research [22–24]. The purpose of TeamSTEPPS® is to improve team structure and team competencies, such as communication, leadership, situation monitoring and mutual support to promote quality, patient safety and the efficiency of healthcare services [25]. These are competencies, referred to in the “Big Five Model of Teamwork” by Salas et al. [24], as essential competencies that affect team performance. The programme provides tools, strategies and measurements to promote team practice in all aspects of healthcare service [25], and uses an implementation strategy based on Kotter’s model of organizational change [26]. Studies of TeamSTEPPS® demonstrate an improvement in patient safety culture [27–29], an improved efficiency in the delivery of patient care and treatment [27, 28, 30] and a reduction in patient complications [31]. Moreover, a correlation between the implementation of TeamSTEPPS® and a reduction in patient mortality has been documented [31].

Despite an increasing awareness of the importance of the teamwork competences, team training in both clinical practice and healthcare education curricula has been implemented to a small extent [32–34]. Teamwork measurements, evaluations and feedback to healthcare personnel may help to promote good team performance [35], with a self-report questionnaire being a common method for measuring teamwork [36]. Questionnaires measuring teamwork competencies are available, although evidence of psychometric validity is missing for most of them [35, 37]. The TeamSTEPPS® Teamwork Perception Questionnaire (T-TPQ), developed by the American Institutes for Research [38] on behalf of the AHRQ as a part of the TeamSTEPPS® package, has been shown to be valid [38, 39]. The T-TPQ measures an individual’s perception of group-level teamwork skills and behaviour within hospital units or departments. The questionnaire includes the five core competencies of teamwork with the following dimensions: team structure, leadership, situation monitoring, mutual support and communication. The T-TPQ measure has shown a good internal consistency (Cronbach’s alpha of 0.88 to 0.96) in previous studies [38, 39]. The questionnaire can be administrated for various purposes, either as assessing health personnel’s perceptions of teamwork, as a part of a site assessment to define training needs in organizations or as a tool to evaluate the effectiveness of TeamSTEPPS® training [38]. The T-TPQ questionnaire has been translated into other languages and adapted to a few cultural contexts [40, 41]. In the context of implementing TeamSTEPPS® in a Norwegian hospital, there was a need to assess teamwork skills and behaviour with a validated, culturally adapted tool in Norwegian. Additionally, there is a need for studies outside US to confirm and test the questionnaire, as well as its relevance to healthcare personnel in other countries. AHRQ gave its permission to translate the questionnaire to Norwegian. The present paper contributes to further teamwork research by addressing how to adapt, refine and evaluate the feasibility of foreign teamwork assessments to national, non-English-speaking healthcare environments. However, to make conclusions about the conceptual and equivalence to the original questionnaire in order to achieve a valid, reliable and culturally sensitive measure, psychometric testing is required [42].

## Method

### Aim

The aim of the study was to translate and cross-validate the T-TPQ into Norwegian, and to test the questionnaire for psychometric properties among Norwegian healthcare personnel.

### The questionnaire

T-TPQ consists of 35 items divided into the five teamwork dimensions: Team Structure, Leadership, Situation Monitoring, Mutual Support and Communication. Each dimension includes seven items on a five-point Likert scale, from 5 = strongly agree with the statement to 1 = strongly disagree with the statement. Each dimension of T-TPQ is calculated to a total sum score or to an average score [38].

### Translation of T-TPQ

The translation followed a model of back-translation inspired by Brislin [43] in a process described in the following five steps:

1. **Forward translation** of the T-TPQ into Norwegian by a professional bilingual translator with Norwegian as his/her native language.
2. **Reviewing** of the translated version by a team [44], including expert groups of nurses and the members of the research team to obtain cross-cultural equivalence: i) A group of three nurses with expert knowledge in the field of teamwork relating to patient safety reviewed the translated version in collaboration with the members of the research team; ii) Five nurses with experience from clinical practice were consulted to help confirm the cultural relevance of the concepts used with regard to a Norwegian healthcare setting. This step generated some semantic and conceptual changes, and resulted in a preliminary initial translated version.
3. **Back-translation** by a second professional bilingual translator with English as his/her native language, who was blinded to the original English version.
4. **Comparison** of the back-translated version and the original version by members of the research team. In this step, only minor inconsistencies were discovered, thereby resulting in some minor revisions.
5. **Pilot testing** of the translated version. To strengthen both sematic and content equivalence [42], the translated version was pilot-tested by 20 healthcare personnel: 11 registered nurses (RNs), three assistant nurses (AN) and six physicians recruited from a hospital. Each participant made comments on items they found unclear [45]. They subsequently gave a response on a scale from 1 to 5 as to whether the items in the questionnaire were relevant, precise, well-articulated and understandable. This last step generated some semantic and conceptual changes, and resulted in the final translated Norwegian version (see Additional file 1).

### Study design, setting, sample and data collection

**Fig. 1:**
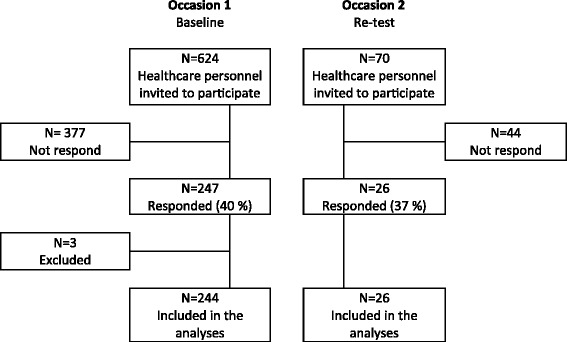
Flowchart of the study sample and data collection

The study utilized a cross-sectional design, and was carried out at two hospitals (Hospital A and Hospital B) in two hospital trusts in Norway. The target population was frontline healthcare personnel (physician, registered nurse (RN), assistant nurse (AN), midwife, physiotherapist and occupational therapist). A survey with a coded paper version of the T-TPQ was carried out on two occasions during the period from October to December 2015 (Fig. 1).

Firstly, the questionnaire was distributed to all healthcare personnel (*n* = 624) employed in medical (Hospital A and Hospital B), gynecological/obstetrical, surgical, intensive care, anesthesia and emergency units (Hospital A). Two reminders were sent. In total, 247 healthcare personnel (40%) responded to the T-TPQ. Three participants with incomplete data (< 50% scores) were deleted (*n* = 244). Secondly, 2 weeks after the completion of the first data collection, the questionnaire was distributed to 70 participants randomly selected from those who responded on the first occasion. Twenty-six healthcare personnel (37%) completed the T-TPQ. The distribution of different healthcare personnel professions in the study sample is shown in Table 1.

**Table 1:** Distribution of healthcare personnel professions in the study sample

	Occasion 1			Occasion 2		
	Invited N = 624	Included *N* = 244	%	Invited *N* = 70	Included *N* = 26	%
Physician	110	11	(4.5)	2	0	–
Registered nurse	405	171	(70.1)	52	19	(73.1)
Midwife	24	13	(5.3)	3	0	–
Assistant nurse	59	27	(11.0)	9	4	(15.4)
Physiotherapist	19	16	(6.6)	3	2	(7.7)
Occupational Therapist	7	6	(2.5)	1	1	(3.8)

### Data analysis

The data were analysed using SPSS version 23 and SPSS AMOS version 23. Descriptive statistics were used to describe sample characteristics and the mean score and standard deviation for each teamwork dimension and single item. A confirmatory factor analysis (CFA) (Model 1) was conducted to test the factor structure of the T-TPQ [46]. Fourteen missing scores distributed among 10 participants were replaced by each participant’s mean score in the relevant dimension [47]. The purpose of a CFA is to test explicit hypotheses about the measure’s dimensionality, and is recommended to be used, e.g., to test whether a factor structure is comparable for different versions of an instrument [48]. This is particularly important with questionnaires that have been translated and/or culturally adapted [48]. Post-hoc modifications (Model 2) were made in accordance with a study by Keebler et al. [39], who examined the construct validity (CFA) of the original English-language version of T-TPQ. To assess the strength of each model, the three fit indexes: the Root Mean Square Error of Approximation (RMSEA), the Tucker-Lewis Index (TLI) and the Comparative Fit Index (CFI) were used. RMSEA represents an absolute fit index [48], and takes into account the error estimates in the population. RMSEA is accepted as the best estimation of how well the model with unknown but optimally chosen parameters values fit the population covariance matrix if it was available ([49], p. 80, 46). For RMSEA, cutoff values close to 0.06 indicate a good fitting model [50], with values as high as 0.08 representing reasonable errors of approximation in the population [49]. TLI and CFI representing indexes of comparative fit [48]. These indexes compare the chi-square values for the hypothesized model with that from a null model, in which all of the variables are uncorrelated, thus having a large chi-square value indicative of a poor fit [48]. For both indexes, cutoff values close to 0.95 offer evidence of a good model fit [50]. A Pearson correlation coefficient was carried out to test the independence of the teamwork construct. The reliability was assessed by Cronbach’s alpha to establish internal consistency for the teamwork dimensions, with a value above 0.70 considered to indicate an acceptable level [51]. An Intraclass Correlation Coefficient (ICC) with the Two-Way Random model was used for test-retest reliability [42].

## Results

The construct validity of the translated T-TPQ was verified through a CFA index standard. The result indicated that each set of seven items that were supposed to accompany each teamwork dimension represent that specific construct. Model 1 showed a reasonable fit with the data (χ^2^ (df) 1180.37 (550), *p* < 0.001, RMSEA = 0.069, TLI = 0.819, CFI = 0.833). The post-hoc modifications according to Keebler et al. [39] referred to four sets of items with high modification indexes, within three of the five dimensions to improve the fit of the model. This included items 12 and 13 under Leadership, items 22 and 23 under Mutual Support, items 26 and 27 under Mutual Support, and items 29 and 31 under Communication, which resulted in the final model (Model 2). This model showed an acceptable fit with the data (χ^2^ (df) 969.46 (546), *p* < 0.001, RMSEA = 0.056, TLI = 0.878, CFI = 0.888) (Table 2).

**Table 2:** CFA fit indices for Model 1 and Model 2 (N = 244)

	CFA index Standard [49].	Model 1	Model 2 (Final model)
χ^2^ (df)	–	1180.37 (550), p < 0.001	969.46 (546), *p* < 0.001
RMSEA	<0.08	0.069	0.056
TLI	>0.95	0.819	0.878
CFI	>0.95	0.833	0.888

CFA = Confirmatory Factor Analysis, RMSEA = Root Mean Square Error of Approximation [49]

The inter-correlation test of the five-teamwork dimensions ranged from 0.52 to 0.71 (Table 3). The internal consistency of the T-TPQ with Cronbach’s alpha demonstrated values from 0.786 to 0.844 on T-TPQ’s five dimensions. The test-retest reliability revealed ICC scores from 0.672 to 0.852 (Table 4). The mean scores and standard deviations for the five teamwork dimensions and the items are shown in Table 5.

**Table 3:** Summary of reliability and correlation for the T-TPQ dimensions (N = 244)

Dimensions	Cronbach’s alpha	Leadership	Situation Monitoring	Mutual Support	Communication
Team Structure	0.786	0.58*	0.67*	0.66*	0.71*
Leadership	0.842		0.58*	0.54*	0.52*
Situation Monitoring	0.826			0.70*	0.68*
Mutual Support	0.844				0.66*
Communication	0.806				

*p < 0.001

**Table 4:** Test-retest reliability (N = 244)

T-TPQ dimensions	^a^ICC (95% Confidence Interval)	F Test Value	*p*
Team Structure	0.819 (0.596–0.919)	5.515	0.001
Leadership	0.852 (0.669–0.934)	6.746	0.001
Situation Monitoring	0.672 (0.269–0.853)	3.052	0.004
Mutual Support	0.761 (0.467–0.893)	4.182	0.001
Communication	0.780 (0.510–0.901)	4.551	0.001

^a^ICC *Intraclass Correlation Coefficient*. Two-Way Random

**Table 5:** Mean scores and standard deviations for T-TPQ items and dimensions

Teamwork dimensions and items		Items Statistics	
		Mean	(SD)
Team Structure		3.96	(0.49)
1.	The skills of staff overlap sufficiently so that work can be shared when necessary	3.85	(0.80)
2.	Staff are held accountable for their actions.	3.88	(0.76)
3.	Staff within my unit share information that enables timely decision making by the direct patient care team.	4.02	(0.64)
4.	Staff within my unit share information that enables timely decision making by the direct patient care team.	3.72	(0.79)
5.	Staff understand their roles and responsibilities.	4.16	(0.61)
6.	My unit has clearly articulated goals.	4.01	(0.84)
7.	My unit operates at a high level of efficiency.	4.04	(0.69)
Leadership		3.81	(0.62)
8.	My supervisor/manager considers staff input when making decisions about patient care.	3.99	(0.68)
9.	My supervisor/manager provides opportunities to discuss the unit’s performance after an event.	4.03	(0.83)
10.	My supervisor/manager takes time to meet with staff to develop a plan for patient care.	3.68	(0.88)
11.	My supervisor/manager ensures that adequate resources (e.g., staff, supplies, equipment, information) are available.	3,66	(0.89)
12.	My supervisor/manager resolves conflicts successfully.	3.71	(1.00)
13.	My supervisor/manager models appropriate team behavior.	3.72	(1.01)
14.	My supervisor/manager ensures that staff are aware of any situations or changes that may affect patient care.	3.87	(0.68)
Situation Monitoring		3.93	(0.47)
15.	Staff effectively anticipate each other’s needs	3.65	(0.70)
16.	Staff monitor each other’s performance.	3.59	(0.76)
17.	Staff exchange relevant information as it becomes available.	4.04	(0.66)
18.	Staff continuously scan the environment for important information	4.03	(0.67)
19.	Staff share information regarding potential complications (e.g., patient changes, bed availability).	4.29	(0.56)
20.	Staff meets to reevaluate patient care goals when aspects of the situation have changed.	4.05	(0.60)
21.	Staff correct each other’s mistakes to ensure that procedures are followed properly.	3.86	(0.72)
Mutual Support		3.92	(0.52)
22.	Staff assist fellow staff during high workload.	4.24	(0.60)
23.	Staff request assistance from fellow staff when they feel overwhelmed.	4.14	(0.64)
24.	Staff caution each other about potentially dangerous situations.	4.16	(0.61)
25.	Feedback between staff is delivered in a way that promotes positive interactions and future change.	3.86	(0.75)
26.	Staff advocate for patients even when their opinion conflicts with that of a senior member of the unit.	3.81	(0.87)
27.	When staff have a concern about patient safety, they challenge others until they are sure the concern has been heard.	3.95	(0.71)
28.	Staff resolve their conflicts, even when the conflicts have become personal.	3.32	(0.84)
Communication		3.91	(0.49)
29.	Information regarding patient care is explained to patients and their families in lay term.	3.95	(0.60)
30.	Staff relay relevant information in a timely manner.	3.92	(0.70)
31.	When communicating with patients, staff allow enough time for questions	3.66	(0.89)
32.	Staff use common terminology when communicating with each other.	3.98	(0.63)
33.	Staff verbally verify information that they receive from one another.	3.85	(0.68)
34.	Staff follow a standardized method of sharing information when handing off patients.	3.88	(0.85)
35.	Staff seek information from all available sources.	4.10	(0.68)

## Discussion

The aim of this study was to translate and cross-validate the T-TPQ into Norwegian, and test the questionnaire for psychometric properties among healthcare personnel. The original questionnaire was developed in the US with a predefined cultural group in mind [48]. However, the use of formally validated and established instruments has the advantage of building a cross-cultural knowledge for which findings can be compared [52]. The Norwegian version of the questionnaire may contribute to improved evidence, knowledge and awareness of teamwork competencies in Norwegian healthcare. The T-TPQ questionnaire may serve as an alternative or complementary measure of teamwork behaviours. Keebler et al. [39] suggest using the questionnaire in healthcare organizations that have implemented TeamSTEPPS®, or similar programmes for improving team training, implementation and sustainment. In Norway, interprofessional teamwork has gained more of a focus in recent years, although no special programmes such as TeamSTEPPS® have thus far been developed and implemented in health care.

There are challenges associated with the translation of a questionnaire [48]. Cross-cultural validity is one type of construct validity [53], and concerns “the degree to which the performance of the items on a translated or culturally adapted instrument are an adequate reflection of the performance of the items of the original version of the instrument” ([53], p. 243). The cross-cultural validity was ensured by a thorough five-step process of translation and back-translation, followed by a pilot testing of the translated version [43]. A challenge in the process was shifting the focus from a simple word for word translation of the questionnaire to its adaption to Norwegian healthcare culture with references to conceptual meaning and linguistic structure. Even though healthcare personnel in Norway work in teams and the concept of teamwork is used in healthcare, healthcare personnel have a light awareness of the core competencies of teamwork [21, 54]. There is still no consensus about a single model or definition of teamwork that can be expected to accommodate every aspect of teamwork within a specific healthcare specialty [8]. Furthermore, teamwork competencies have not been addressed in a systematic way by healthcare systems in general [55], and knowledge related to teamwork has probably been more practical and tacit. In this context, it was important to achieve a translation that gave meaning for the healthcare personnel, but at the same time was true to the English version. The pilot testing with a sample of healthcare personnel was important to ensure that the items made sense in a clinical setting.

Another aspect of construct validity is structural validity that refers “to the extent to which the structure of a multi-item scale adequately reflects the hypothesized dimensionality of the construct being measured” ([56], p. 318). The CFA in this study was performed in accordance with the US T-TPQ validation study [39], with post hoc modifications to improve the fit due to high modification indexes in four sets of items within three of the five dimensions. These items contain a highly similar content which would therefore lead to correlated errors [39]. However, no changes were made in the original English-language version of T-TPQ developed by the American Institutes for Research [38]. In this study, the result from the post hoc modification (Model 2) exhibited an RMSEA index of 0.056, which indicates a reasonable fit [49]. Nonetheless, the two indexes, TLI = 0.88 and CFI = 0.89, are slightly below the values that offer a good evidence of model fit. However, RMSEA is recognized as the most informative and robust criteria in covariance structure modelling [46, 49, 57, 58]. The study by Keebler et al. [39] exhibited better values on the two indexes TLI (0.94) and CFI (0.94) to a certain extent while the RMSEA (0.057) was almost the same as in our study. The Korean study by Hwang, Ahn [41] reported a more modest RMSEA (0.067) value. In their study, only nurses participated, which may have had an impact on the result. CFA works best when the sample is large, which enables stabile parameter estimates [48]. In the same study by Keebler et al. [39] the sample was large, 1700 staff members from US Army medical facilities were included, which could be an explanation for the better outcome. In this study, 244 participants provided seven cases for each parameter, which are in line with the recommendations of 5–10 [59]. A larger sample may have resulted in a CFA model with a better fit with data [48].

The ICC is the preferred reliability parameter for test-retest reliability, also called stability or reproducibility [56]. A review of the literature showed that the criteria for acceptable ICC values vary from one expert to another, so the standard for reliability might vary according to the situation. Polit [60] advises developers of new measures to aspire to test-retest reliabilities of 0.80 or higher. In this study, the ICC varied from 0.672–0.852. A problem for the test-retest reliability of instruments used in healthcare may be linked to the fact that healthcare personnel’s perceptions do change over time, and sometimes even over a short period. Healthcare personnel’s attitude, knowledge and skills can be modified by experiences between the test and the retest, and change would make a measure less reliable than it actually is [56].

Some issues regarding the sample and response rate of the study should be noted. The analysis was undertaken with a sample from the population for whom the measure is intended, but only two hospitals were included. Moreover, the response rate was low, with only 10% of the physicians responding to the questionnaire. Asch et al. [61] reported that surveys of physicians had a lower response rate than surveys of other healthcare personnel, while Cook et al. [62] did not find any differences between healthcare professions. However, surveys of physicians had a decrease in response rates from 1995 to 2005. We do not know whether all subjects were actually eligible for the study, and the dropouts may be associated with staff turnover, sick leave, and working schedules. Moreover, the term *teamwork* still has different meanings to various healthcare professions and the lack of a shared understanding of team structure, team roles and tasks in connection with a patient’s care team [8, 63], may have influenced the motivation to respond to the questionnaire. The perceptions of interprofessional teamwork may be influenced by professional role identities. Aase et al. [64] found nursing students were more likely to share the responsibility than medical students, who regarded taking responsibility at an individual level. Dropout analyses with background variables, such as age and sex, between the responders and the non-responders were not performed because we did not have access to this data. A low response rate might involve a risk of bias, which may affect the external validity of the study [56]. Based on these limitations, it is important to carry out additional studies that include more participants and, above all, motivate more physicians to respond. The internal dropout was low, with only 14 missing scores (less than 1%), distributed over 10 participants. To satisfy the requirements for running a CFA, missing substitutes were manually conducted using the “case mean substitution technique” [47].

## Conclusions

The Norwegian translated version of T-TPQ was considered to be acceptable regarding validity and reliability for measuring individual healthcare personnel’s perception on group level teamwork at the front line within their unit. However, it needs to be further tested, preferably in a larger sample and different clinical settings. A further psychometric testing of the Norwegian T-TPQ questionnaire is therefore required to establish the psychometric property and a multisite study with a range of variation among different types of healthcare systems across several healthcare settings and professionals would be desirable. The T-TPQ highlights opportunities to identify areas for teamwork improvement as part of the promotion of patient safety. Healthcare organizations implementing TeamSTEPPS® programme may use the T-TPQ for a continuous evaluation of team training and the sustainability of the teamwork skills.

## Additional file

Additional file 1: The Norwegian-language version of TeamSTEPPS® Teamwork perception Questionnaire. (DOCX 19 kb)

## Abbreviations

AHRQ: Agency for Healthcare Research and Quality
AN: Assistant nurse
CFA: Confirmatory factor analysis
CFI: Comparative Fit Index
ICC: Intraclass Correlation Coefficient
RMSEA: Root Mean Square Error of Approximation
RN: Registered nurse
TeamSTEPPS®: Team Strategies and Tools to Enhance Performance and Patient Safety
TLI: Tucker-Lewis Index
T-TPQ: TeamSTEPPS Teamwork Perception Questionnaire
