# Supplementary material for: Cross-cultural validation and psychometric testing of the Norwegian version of the TeamSTEPPS® teamwork perceptions questionnaire
Source: BMC Health Serv Res. 2017 Dec 2;17:799. doi: 10.1186/s12913-017-2733-y (PMC5712180; doi:10.1186/s12913-017-2733-y)
Supplement: Additional file 1: — The Norwegian-language version of TeamSTEPPS® Teamwork perception Questionnaire. (DOCX 19 kb) [file 12913_2017_2733_MOESM1_ESM.docx]

**Additional file 1**

**The Norwegian-language version of TeamSTEPPS^®^ Teamwork perception Questionnaire (T-TPQ)**

| **Teamstruktur** | |
| --- | --- |
| 1. | Kompetansen til helsepersonellet er tilstrekkelig overlappende slik at enkelte arbeidsoppgaver kan deles på når det er nødvendig. |
| 2. | Helsepersonellet blir holdt ansvarlig for egne handlinger. |
| 3. | Helsepersonellet i enheten deler informasjon på en måte som gjør det mulig for pasientens behandling/pleieteam å ta gode beslutninger i rett tid. |
| 4. | I enheten utnyttes ressursene på en hensiktsmessig måte (eks. bemanning, utstyr, informasjon). |
| 5. | Helsepersonellet har en klar oppfattelse av egne roller og ansvar. |
| 6. | Enheten har klart formulerte mål. |
| 7. | Enheten fungerer på en hensiktsmessig måte. |
| **Ledelse** | |
| 8. | Teamledere vurderer innspill fra helsepersonellet i enheten når det tas beslutninger vedrørende pasientbehandling/pleie. |
| 9. | Min nærmeste leder legger til rette for å diskutere enhetens opptreden etter en hendelse som kunne ha ført til, eller førte til unødig skade hos en pasient. |
| 10. | Teamledere tar seg tid til å møte helsepersonellet i enheten for å utvikle planer for pasientbehandling/pleie. |
| 11. | Min nærmeste leder sørger for at det er tilstrekkelig ressurser tilgjengelig (eks. bemanning, utstyr, informasjon). |
| 12. | Min nærmeste leder håndterer konflikter på en god måte. |
| 13. | Min nærmeste leder er en god rollemodell når det gjelder teamadferd. |
| 14. | Teamledere sørger for at helsepersonellet i enheten er oppmerksomme på situasjoner eller endringer som kan påvirke pasienters behandling og pleie. |
| **Situasjonsovervåking** | |
| 15. | Helsepersonellet er flinke til å forutse hverandres behov. |
| 16. | Helsepersonellet observerer hverandre i utførelse av arbeidsoppgaver. |
| 17. | Helsepersonellet utveksler relevant informasjon så fort den blir tilgjengelig. |
| 18. | Helsepersonellet følger nøye med på alt i omgivelsene rundt pasienten for å innhente viktig informasjon. |
| 19. | Helsepersonellet deler informasjon om potensielle problemer som oppstår (eks. endringer i pasientens tilstand, full avdeling etc.). |
| 20. | Helsepersonellet kommer sammen og revurderer planen for pasientens behandling og pleie når tilstanden og/eller situasjonen har endret seg. |
| 21. | Helsepersonellet korrigerer hverandre slik at enhetens prosedyrer blir fulgt. |
| **Gjensidig støtte** | |
| 22. | Helsepersonellet hjelper hverandre når det er mye å gjøre. |
| 23. | Helsepersonellet spør om hjelp fra kollegaer når de føler at det blir for mye for dem. |
| 24. | Helsepersonellet advarer hverandre om potensielle faretruende situasjoner. |
| 25. | Tilbakemeldinger gis til hverandre på en måte som fremmer positivt samarbeid og som fører til fremtidige endringer. |
| 26. | Helsepersonellet taler pasientens sak, selv når det kommer i konflikt med det som hevdes av en «senior» kollega i enheten. |
| 27. | Når helsepersonellet er bekymret for pasientsikkerheten, sier de tydelig i fra til hverandre og gir seg ikke før de er sikre på at bekymringen er blitt hørt. |
| 28. | Helsepersonellet løser konflikter seg imellom, også når konfliktene er personlige. |
| **Kommunikasjon** | |
| 29. | Informasjon om pasientbehandling/pleie blir formidlet til pasientene og deres pårørende på et forståelig språk. |
| 30. | Helsepersonellet formidler relevant informasjon til pasienter og deres pårørende så fort som mulig. |
| 31. | Når helsepersonellet kommuniserer med pasienten, sørger de alltid for at det er tid og rom for spørsmål. |
| 32. | Helsepersonellet bruker felles terminologi/fagspråk når de kommuniserer med hverandre. |
| 33. | Helsepersonellet gir verbal bekreftelse på mottak av viktig informasjon fra hverandre. |
| 34. | Helsepersonellet følger en standardisert metode for overføring av informasjon ved overlevering av pasienter (eks. vaktskiftet, overflytting). |
| 35. | Helsepersonellet innhenter informasjon fra alle tilgjengelige kilder (eks. pasienten, pårørende, teamet, journal, laboratoriesvar, forskning). |

Translated by Department of Health Science Gjøvik, Norwegian University of Science and Technology, Norway, with permission from US Agency for Healthcare Research and Quality (AHRQ).

**Avklaring av begrep som benyttes i T-TPQ**

**Helsepersonell** er helseprofesjoner som er involvert i den direkte pasientbehandlingen/pleie som en del av et tverrfaglig team (eks. leger, sykepleiere, jordmødre, helsefagarbeidere/hjelpepleiere, barnepleiere, fysioterapeuter og ergoterapeuter).

**Et team** kan defineres som en gruppe på to eller flere enkeltpersoner som er avhengig av hverandre i arbeidet mot et felles mål, og hvor det kreves samordning av innsats og ressurser for å oppnå et felles ønsket resultat og hvor alle deltagere har spesifikke roller eller funksjon. Team har ofte en tidsbegrenset deltagelse [1].

**Teamarbeid** i helsetjenesten beskrives som samspillet mellom to eller flere helsepersonell (team-medlemmer) som arbeider gjensidig avhengig av hverandre for å gi behandling og pleie til pasienter [2].

**Teamledere** er helsepersonell som har et forhåndsbestemt eller situasjonsbetinget ansvar for å lede og koordinere aktivitetene til andre gruppemedlemmer (eks. vakthavende lege, visittansvarlig lege, sykepleiere med gitt ansvar, eller jordmor).

Med **pasientbehandling/pleie** menes: legebehandling, sykepleie, fysioterapi, ergoterapi etc.

**Pasientsikkerhet** defineres som «Vern mot unødig skade som følge av helsetjenestens ytelser eller mangel på ytelser» (Nasjonal kunnskapssenter for helsetjenesten).

**En uønsket hendelse** er en skade som er relatert til klinisk behandling og ikke til komplikasjoner ved et sykdomsforløp. Med klinisk behandling menes alle aspekter av helsetjenester inkludert diagnose, behandling, pleie, systemer og utstyr som brukes for å levere tjenestene. Uønskede hendelser kan være mulig å forebygge eller ikke [3].

**Referanser**

1. Salas E, Dickinson TL, Converce S, Tannenbaum SI. Towards an understanding of teams performance and training. In: Swezey RW, Salas E, editors. Teams: Their training and performance. Norwood, N.J.: Ablex Publ.; 1992. p. 3-29.

2. Oandasan I, Ross Baker GR, Barker K, Bosco C, D’Amour D. Teamwork in healthcare: Promoting effective teamwork in healthcare in Canada. Policy synthesis and recommendations. Ottawa: Canadian Health Services Research Foundation; 2006.

3. WHO. WHO Draft Guidelines for Adverse Event Reporting and Learning Systems. From information to action. World Health Organisation. 2005. http://www.who.int/patientsafety/events/05/Reporting_Guidelines.pdf. Retrieved 2011-02-15.
